# Assesment of Conjunctival Microangiopathy in a Patient with Diabetes Mellitus Using the Retinal Function Imager

**DOI:** 10.4172/2155-9570.1000400

**Published:** 2015-02-20

**Authors:** Nicole Stuebiger, William Smiddy, Jianhua Wang, Hong Jiang, Delia Cabrera DeBuc

**Affiliations:** 1 Bascom Palmer Eye Institute, University of Miami, 900 NW 17th Street, Miami, Florida, 33136, USA; 2 Charite, Universitaetsmedizin Berlin, Campus Benjamin Franklin, University Eye Hospital, Hindenburgdamm 30, 12200 Berlin, Germany

**Keywords:** Diabetes mellitus, Conjunctival microangiopathy, Retinal function imager, Retinal microangiopathy, Optical imaging

## Abstract

Diabetes mellitus (DM) is notorious for causing retinal microangiopathy, but bulbar conjunctival microangiopathy (CM) mirroring the established retinal vessel changes, has also been observed. Recent studies suggest that CM occurs in all DM patients in various degrees depending on disease severity and occur even before non-proliferative retinopathy develops. Thus, CM might provide a means of early detection or even form a basis for early intervention of disease progression in DM patients. Herein we present - to our knowledge for the first time-the feasibility and applicability in diagnostic imaging of CM in a diabetic patient using a commercially available Retinal Function Imager (RFI, Optical Imaging Ltd, Rehovot, Israel).

## Introduction

Patients with Diabetes mellitus (DM) mostly develop changes in the blood flow [1–3], leading to well-known vessel changes in the conjunctiva and in the retina, such as loss of capillaries, microaneurysms, vessel dilation and vascular tortuosity [1,4–8]. Retinal vascular function and morphology measures, such as the blood flow (BF) velocity and capillary perfusion, have been assessed without contrast using the Retinal Function Imager (RFI). The RFI is a fundus camera-based device with an attachment of a specific camera (a 60-Hz, 1024 × 1024-pixel digital camera) that captures reflectance changes as a function of time under stroboscopic illumination (wavelengths between 530 and 590 nm). This device was originally designed to measure the BF velocity directly and noninvasively (without using any contrast agent) in secondary and tertiary retinal arteries and veins and to generate non-invasive capillary perfusion maps (nCPMs) of the retinal microvasculature while using the hemoglobin in the red blood cells as an intrinsic motion-contrast agent [9–11].

Its use to study the conjunctival microvasculature (CM) and its overall BF velocity in healthy subjects was presented for the first time in 2013, opening a venue for analyzing both functional and morphological changes in the eye through the conjunctiva using the RFI device [12]. However, noninvasive capillary perfusion map (nCPM) imaging and BF velocity in a wide field have not been generated previously for the conjunctiva in diabetic patients. Recent studies suggest that CM occurs in all DM patients in various degrees depending on disease severity [4,6]. In addition conjunctival vessels seem to mirror the established vessel changes observed in the diabetic retina, and may even be evident before diabetic retinopathy develops [4–7]. Thus, CM might merit more attention.

This prompted us to investigate the retinal vasculature but also the conjunctival vascular morphology in a DM patient with the RFI device [9–11]. The study was approved by the Institutional Review Board of the University of Miami, Miami, FL, USA. The research adhered to the tenets set forth in the declaration of Helsinki and written informed consent was obtained from the subject.

## Case Report

**Figure 1:**
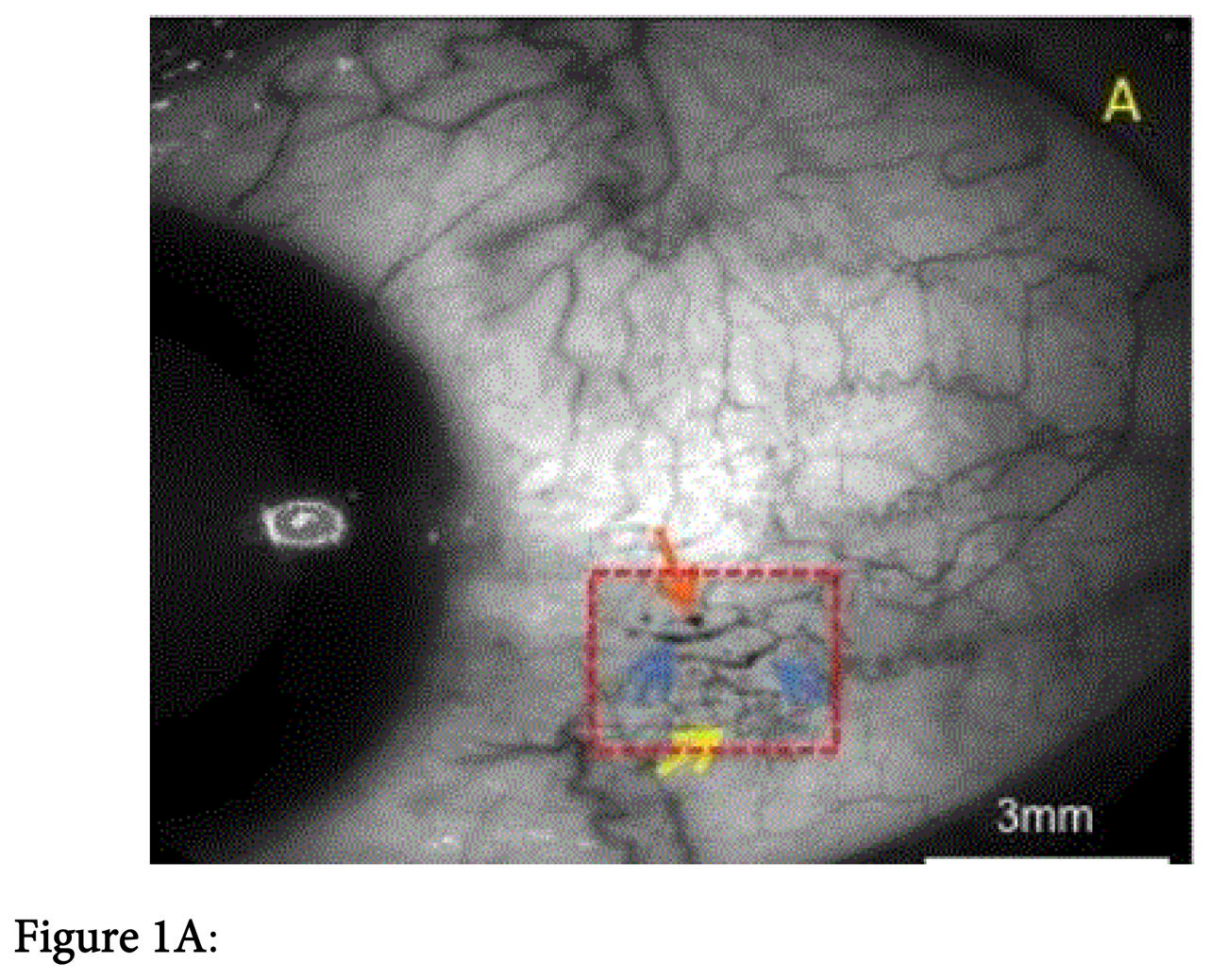

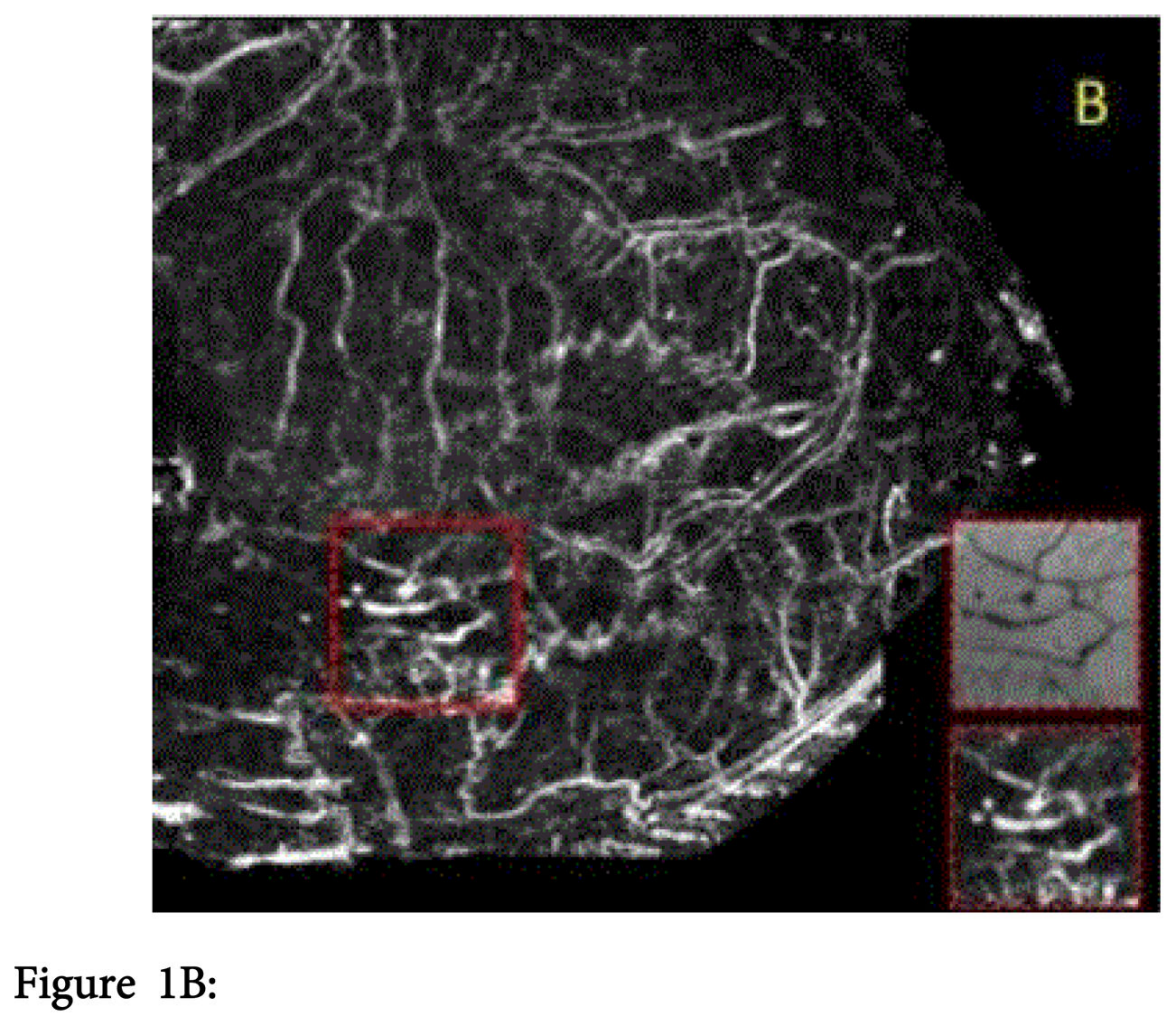

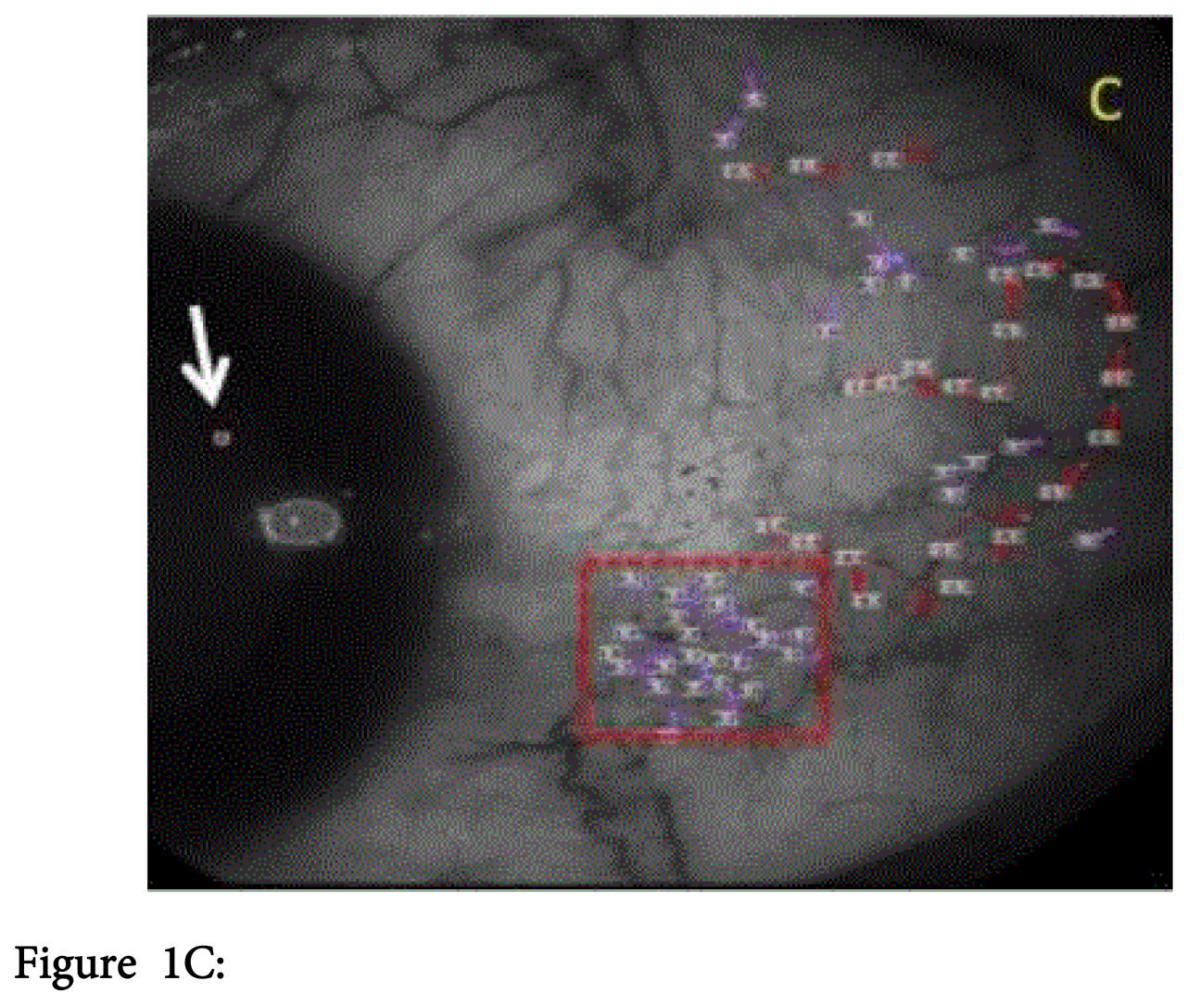

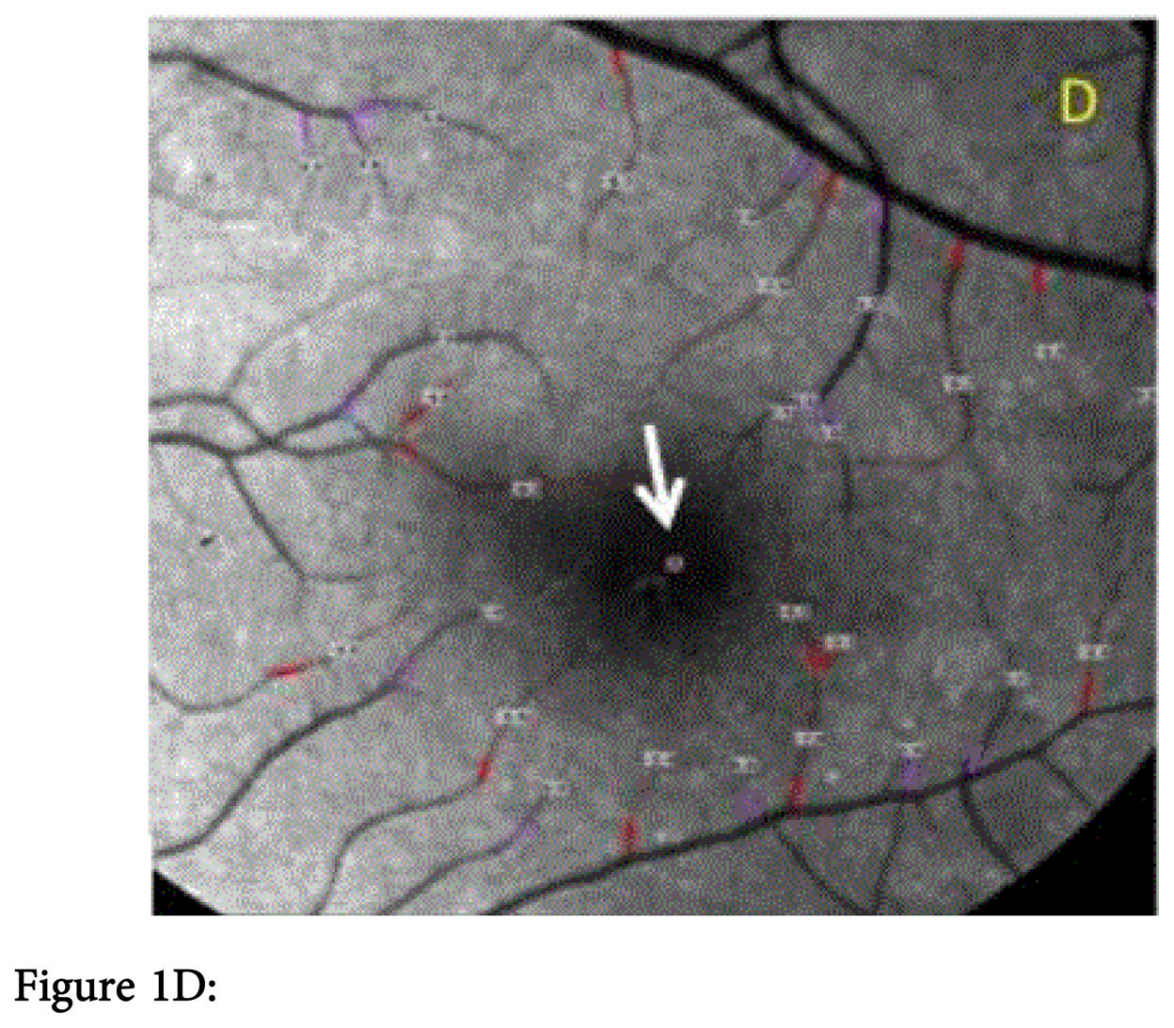

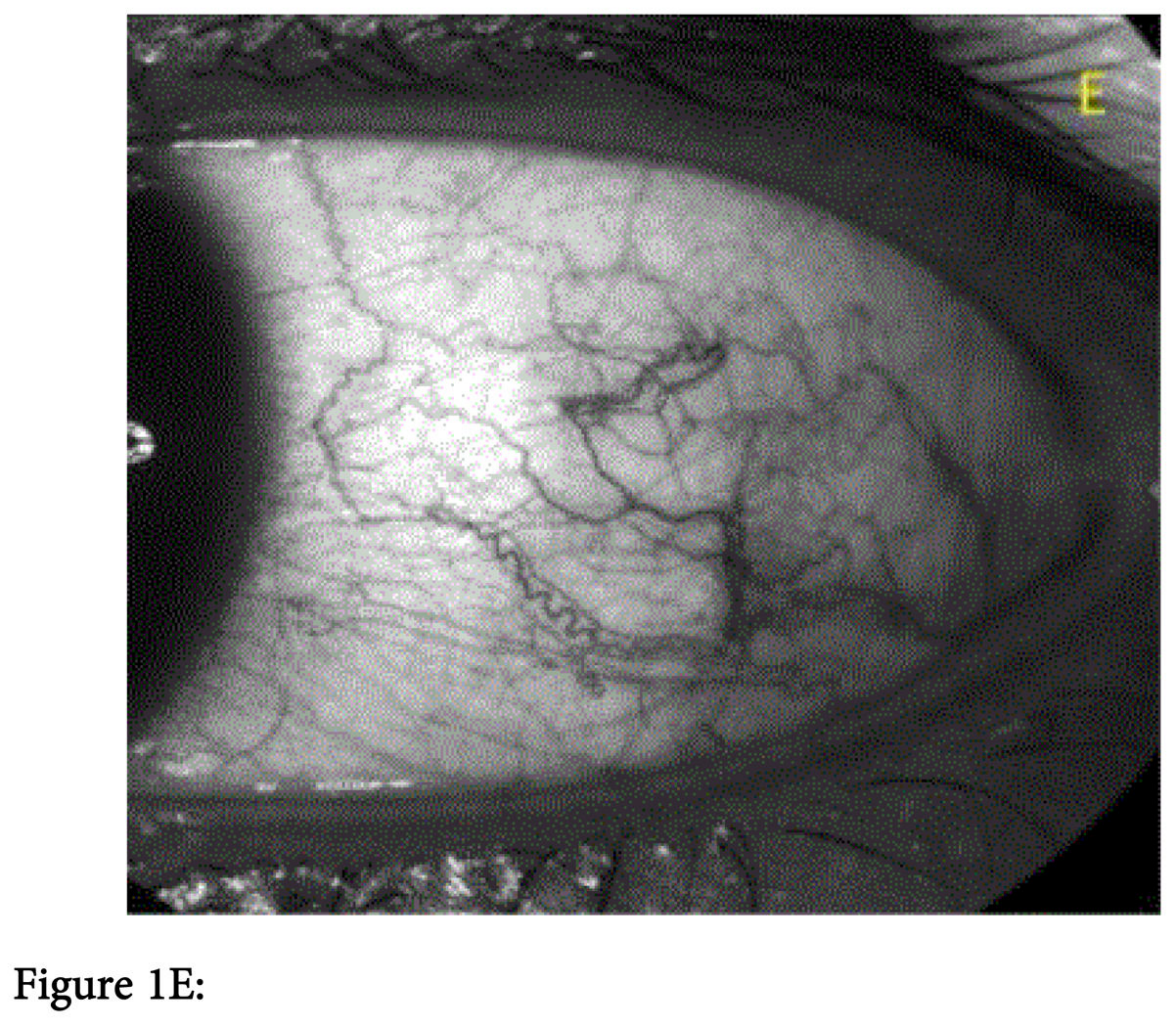

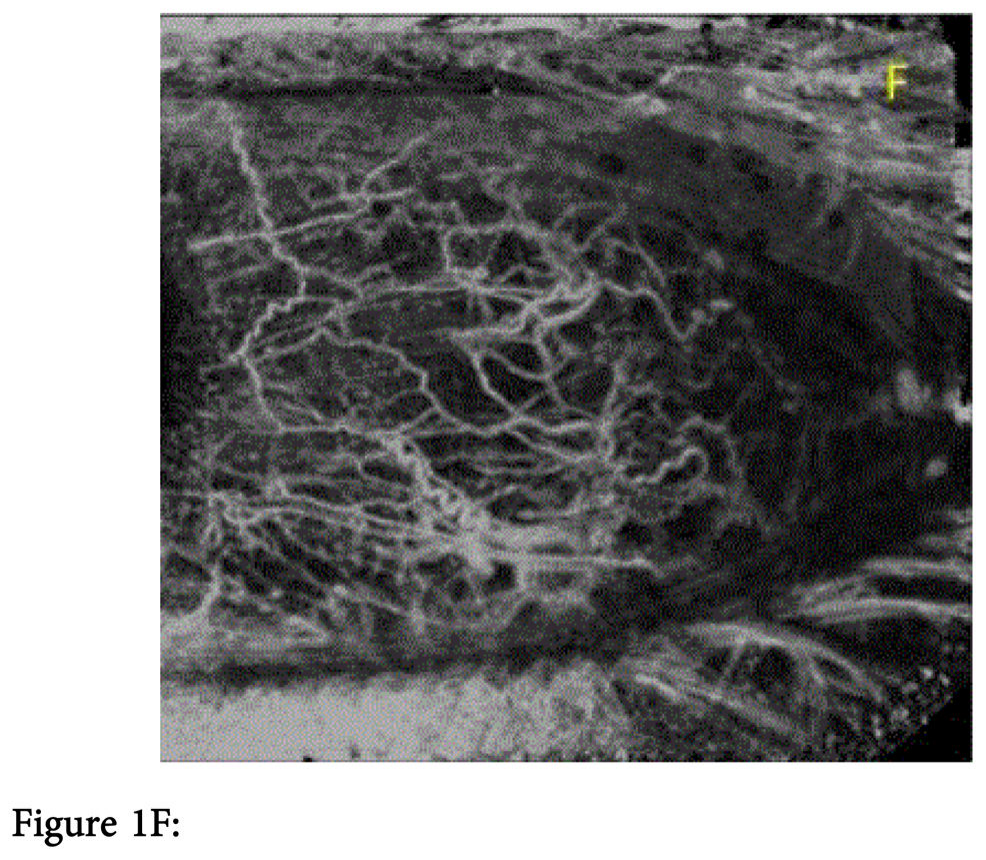
Figure 1A: Bulbar conjunctiva of the left eye imaged in a 50 degrees red-free mode with the RFI. Temporal conjunctiva of the patients left eye with microangiopathy of the conjunctival vessels (red dashed-square): microaneurysms (orange arrow is indicating one of them), vessel dilatation (blue arrows), and vascular tortuosity (yellow arrow). Figure 1B: Bulbar conjunctival capillary perfusion map (nCPM). Note the microvasculature visualization enhancement showing the microvasculature anatomy in detail, otherwise invisible even in the sharpest red-free image (Figure 1A). The microvasculature anatomy appeared unevenly distributed. Figure 1C: Analysis of the BF velocity of the conjunctiva. The positive values with the purple lines are representing the veins, the negative values with the red lines are representing the arteries, the numbers are the BF velocity in mm/s. Values in the area with the red-dashed square are representing the BF data of the area with the vascular abnormalities. The symbol on the cornea (white arrow) marks the direction of the arterial BF. Figure 1D: Analysis of the BF velocity of the central retina imaged in a 20 degrees red-free mode. Note that the positive values with the purple lines are representing the veins, the negative values with the red lines, which indicate BF moving away from the heart, are showing the arteries. The numbers closed to vessel segments outlined are the mean BF velocity in mm/s. The avascular zone was evident in the fovea. The symbol in the fovea (white arrow) is indicating the direction of the arterial BF. Note that no clinical signs of DR are present. Figure 1E: Bulbar conjunctiva of a human healthy’s left eye imaged in a 50 degrees red-free mode with the Retinal Function Imager. Figure 1F: Bulbar conjunctival capillary perfusion map (nCPM) of the same human healthy eye (Figure 1E). Note the microvasculature visualization enhancement showing the microvasculature anatomy in detail, otherwise invisible even in the sharpest red-free images (Figure 1E). The microvasculature anatomy appeared evenly distributed and lower number of blood vessels along with lower degree of complexity of their branching patterns increased density and complexity is evident when compared with the diabetic eye (Figure 1B).

The conjunctival and retinal vasculature was imaged in a 38 years-old black female DM patient without DR with a commercially available RFI device. This patient was a non-smoking female with a history of Type-2 DM (T2DM) since 2009. She was being treated with Metformin 2 × 500 mg per day. Hemoglobin A1C was 5.3% (34 mmol/mol) and glucose was 87 mg/dL. Diastolic blood pressure was slightly elevated (120/90 mmHg). Ocular history included a nasally located pterygium removal in both eyes in 2010 and occasional seasonal allergic eye disease. The eye examination disclosed CM in the temporal area of the DM patient’s left eye (Figure 1A).

All the other findings were within normal limits in both eyes; specifically, there were no clinical signs of acute allergic eye disease or diabetic retinopathy (DR) and the visual acuity was 20/20. The temporal conjunctiva and the retina of the patient’s left eye (Figure 1A–1D) were imaged under normal conditions with the RFI by the same operator. The overall conjunctival and retinal BF velocities and nCPMs were calculated using the proprietary software of the RFI device as an average of selected arteriole and venule segments (Figure 1C and 1D). In addition to the conjunctival BF calculation we analyzed a region of interest (ROI) incorporating the microangiopathy of the conjunctival vessels, (Figure 1A), and compared it to regions without morphometric abnormalities.

When imaging the temporal conjunctival microvasculature with the RFI we could illustrate microaneurysms, vessel dilation, abnormal vessel distribution and vascular tortuosity (Figure 1A) in the patient’s left eye. The nCPMs were successfully acquired and revealed the intricate capillary network structure on the temporal bulbar conjunctiva (Figure 1B). The microvasculature anatomy appeared unevenly distributed, and lower numbers of blood vessels along with lower degree of complexity of their branching patterns were evident when compared with a normal healthy eye (Figure 1A–1B vs. 1E–1F).

The overall BF velocity (Figure 1C) in the temporal conjunctival vessels of the diabetic patient was 0.68 ± 0.31mm/s compared to 0.86 ± 0.08 mm/s in healthy controls [12] (p=0.61). Compared to regions without morphometric abnormalities (0.62 ± 0.25 mm/s), the BF velocity was increased to 1.01 ± 0.37 mm/s (p=0.007) in the ROI incorporating the conjunctival vessel dilatation, microaneurysms, and more packing of tortuous vessels (Figure 1A). Retinal BF velocity in the same eye (Figure 1D) disclosed increased arterial BF velocity of 5.16 ± 1.66 mm/s compared to 4.19 ± 0.99 mm/s in healthy controls [13] (p=0.03), whereas the venous BF calculation revealed values similar to healthy controls [13] (2.99 ± 0.85mm/s versus 3.03 ± 0.59 mm/s; p=0.3).

## Discussion

This case report is the first to demonstrate the feasibility of optical imaging using the RFI for obtaining high-resolution nCPMs and providing the BF velocity of the bulbar conjunctiva vessels in a diabetic patient. The DM patient in this study had CM with overall conjunctival BF velocity comparable to that previously reported in healthy controls except for a significant increase of the BF velocity in the altered conjunctival area due to marked vessel dilatation (Figure 1A). These conjunctival vessel alterations were clinically visible and clearly imagined despite the absence of clinically discernible retinal microangiopathic findings (Figure 1D). The retinal BF velocity calculated with the RFI’s custom-built software RFI in the same eye detected increased BF velocity in the retinal arteries, while the venous velocity revealed no significant alteration compared to healthy controls. Increased BF velocity in the retinal arteries has been described in patients with pre-retinopathy [13]. The most plausible physiologic explanation for this scenario is that retinal arteries widen in response to impaired capillary perfusion, while retinal venous diameters remain relatively constant [13].

The conjunctival vessel alterations observed in this DM patient may have been influenced by the allergic eye disease or the slightly elevated diastolic blood pressure because both could lead to conjunctival vessel alterations [5,14]. While allergic eye disease induce vessel dilation [14], it was unlikely in our patient since allergic symptoms were not present while doing the RFI examination. Elevated blood pressure could also be responsible for CM [5,8], in particular conjunctival tortuous vessels have been linked with hypertension [5]. But an influence in our patient is more than improbably - on the one hand only the diastolic blood pressure was slightly elevated to 90 mmHg and on the other hand recent studies demonstrated that diabetic CM predominates in those patients with both diabetes and elevated blood pressure [5].

In the United States more than 23 million people suffer from diabetes mellitus, an increasingly prevalent disorder. Extensive research has been conducted to determine effective methods of detection and treatment of diabetes mellitus and its attendant ocular complications. Studying methods to detect these complications in their earliest stages may inspire development of early treatment strategies before clinical signs are apparent, mitigating irreversible damage to the microvasculature. This is essential in diabetic patients because early treatment, e.g. intensive glycemic control or pancreas transplantation, of diabetic vessel alterations has been shown to retard both their development and progression in adults; in childhood, if detected early, the vessel changes could even be reversed [7,15].

In conclusion because recent studies suggest that conjunctival vessels seem to mirror the established vessel changes observed in the diabetic retina, and may even be evident before diabetic retinopathy develops [4–7], CM might merit more attention. This study demonstrates a possible role for the RFI as an in vivo platform to monitor these conjunctival vessel alterations. A pilot study with a larger amount of patients should be initiated to figure out if there might be an indication for the presence of a time window for early intervention in DM patients with conjunctival microangiopathy before non-proliferative DR develops. It may also allow new insights on the pathogenesis of vascular changes in the diabetic eye.
